# Screen-Printed PVDF Piezoelectric Pressure Transducer for Unsteadiness Study of Oblique Shock Wave Boundary Layer Interaction

**DOI:** 10.3390/mi15121423

**Published:** 2024-11-27

**Authors:** Bei Wang, Cosimo Corsi, Thomas Weiland, Zhenyu Wang, Thomas Grund, Olaf Pohl, Johannes Max Bienia, Julien Weiss, Ha Duong Ngo

**Affiliations:** 1 Department of Microsystem Technology, University of Applied Sciences Berlin, 12459 Berlin, Germany; thomas.weiland@htw-berlin.de (T.W.); olaf.pohl@htw-berlin.de (O.P.); johannes.bienia@htw-berlin.de (J.M.B.); 2 Department of Aerodynamics Engineer, Technical University of Berlin, 10587 Berlin, Germany; cosimo.corsi@tu-berlin.de (C.C.); thomas.grund.1@tu-berlin.de (T.G.); julien.weiss@tu-berlin.de (J.W.); 3 Deye Neue Enerige Gmbh, Nordostpark 98-102, 90411 Nürnberg, Germany; zhenyu.wang@outlook.de; 4 Ningbo Deye ESS Technology Co., Ltd., No.568, South Rixian Road, Binhai Economic Development Zone, Cixi, Ningbo 315311, China

**Keywords:** supersonic, shock wave/boundary layer interaction (SWBLI), PVDF, piezoelectric pressure transduce, FEM simulation, dynamic response in frequency domain, crosstalk effect, wall pressure, unsteadiness

## Abstract

Shock wave boundary/layer interactions (SWBLIs) are critical in high-speed aerodynamic flows, particularly within supersonic regimes, where unsteady dynamics can induce structural fatigue and degrade vehicle performance. Conventional measurement techniques, such as pressure-sensitive paint (PSP), face limitations in frequency response, calibration complexity, and intrusive instrumentation. Similarly, MEMS-based sensors, like Kulite^®^ sensors, present challenges in terms of intrusiveness, cost, and integration complexity. This study presents a flexible, lightweight polyvinylidene fluoride (PVDF) piezoelectric sensor array designed for high-resolution wall-pressure measurements in SWBLI research. The primary objective is to optimize low-frequency pressure fluctuation detection, addressing SWBLI’s need for accurate, real-time measurements of low-frequency unsteadiness. Fabricated using a double-sided screen-printing technique, this sensor array is low-cost, flexible, and provides stable, high-sensitivity data. Finite Element Method (FEM) simulations indicate that the sensor structure also has potential for high-frequency responses, behaving as a high-pass filter with minimal signal attenuation up to 300 kHz, although the current study’s experimental testing is focused on low-frequency calibration and validation. A custom low-frequency sound pressure setup was used to calibrate the PVDF sensor array, ensuring uniform pressure distribution across sensor elements. Wind tunnel tests at Mach 2 verified the PVDF sensor’s ability to capture pressure fluctuations and unsteady behaviors consistent with those recorded by Kulite sensors. The findings suggest that PVDF sensors are promising alternatives for capturing low-frequency disturbances and intricate flow structures in advanced aerodynamic research, with high-frequency performance to be further explored in future work.

## 1. Introduction

**Figure 1:**
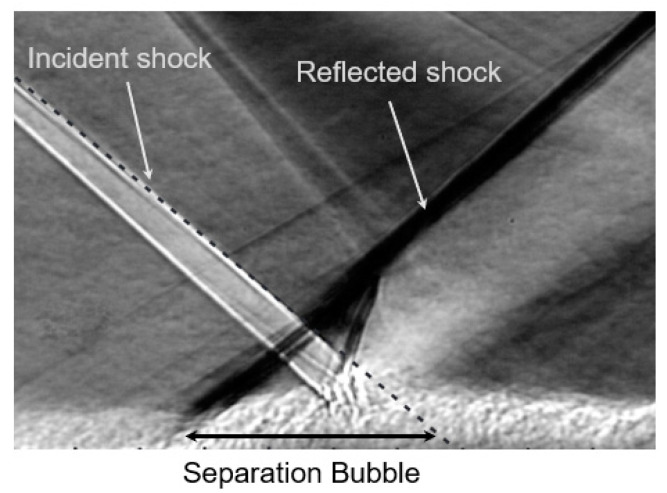
Schlieren image of the flow field of the shock wave/boundary layer interaction [3].

A shock wave/boundary layer interaction (SWBLI) occurs in high-speed aerodynamic flows, particularly in supersonic and hypersonic regimes. This interaction is influenced by factors such as shock wave strength, the angle of incidence on the boundary layer, Reynolds number, and boundary layer characteristics. These factors lead to abrupt changes in flow properties, resulting in shock wave formation and regions with pressure and temperature gradients. Near solid surfaces, a thin boundary layer forms, characterized by gradual changes in flow properties from the surface to freestream conditions [1,2,3]. When the shock wave impinges on the boundary layer, interactions occur, resulting in significant flow modifications, as shown in Figure 1. These modifications can cause the boundary layer to either separate from the surface or undergo substantial thickening or thinning [2,3]. Changes in the boundary layer induce unsteady behaviors, such as low-frequency oscillations, flow separations, turbulence generation, and pressure fluctuations [2,3,4,5]. These unsteady phenomena can lead to vibrations, buffeting, and even structural fatigue in aircraft and other high-speed vehicles [6,7]. Thus, understanding and predicting the unsteadiness caused by shock wave/boundary layer interactions is essential for designing aerodynamically stable and structurally robust systems.

For many years, researchers have employed both experimental measurements and computational flow simulations to observe and analyze interactions, visualize flow fields, and obtain qualitative information. Optical methods like schlieren imaging capture refractive index changes due to density gradients but are limited by calibration and small density differences [8]. Particle Image Velocimetry (PIV) visualizes flow fields by tracking tracer particles, although its accuracy is affected by factors such as particle size, lighting, and background noise [9,10,11,12].

Wall-pressure measurements are an essential technique in both ground and flight tests for understanding SWBLIs. These measurements help identify high- and low-pressure regions, such as separation bubbles, validate simulations, and capture the frequency characteristics of unsteadiness [13,14,15,16,17]. Wall-pressure measurements directly assess pressure distribution, offering a more accurate depiction of unsteadiness associated with SWBLIs. However, the use of pressure-sensitive paint (PSP) or Kulite^®^ sensors (Kulite Semiconductor Products, Inc., One Willow Tree Road, Leonia, NJ, USA) is limited by factors like installation, structure, and cost, making them unsuitable for large-area sensing and integration [13,18].

Polyvinylidene Fluoride (PVDF), a thermoplastic fluoropolymer, has been valued for decades due to its flexibility and stretchability, enabling PVDF sensors to adapt to various shapes and surfaces [19,20,21,22,23,24]. These sensors are highly sensitive and structurally simple, making them effective for detecting mechanical stimuli such as pressure, strain, and vibration [25,26,27].

In the study of SWBLIs, low-frequency unsteadiness often dominates critical flow behaviors, including flow separation and recirculation bubble formation, making accurate low-frequency pressure measurements essential. To address this need, this study develops a flexible polyvinylidene fluoride (PVDF) piezoelectric sensor array optimized specifically for low-frequency pressure fluctuation detection in SWBLI environments. Our design and processing methods differ significantly from traditional MEMS sensors and existing approaches [28,29]. The PVDF sensor array, fabricated using a double-sided screen-printing technique, provides a low-cost, lightweight, and flexible solution that can adapt to varying surface shapes while delivering high-sensitivity measurements.

Finite Element Method (FEM) simulations indicate that the PVDF sensor array has potential for a stable high-frequency response up to 300 kHz, demonstrating high-pass filter characteristics with minimal signal attenuation at higher frequencies. Although the current experimental testing focuses on low-frequency calibration and validation to meet the primary needs of SWBLI research, the sensor’s design and simulation results provide a foundation for exploring its high-frequency response in future studies. This approach allows us to address immediate research needs in low-frequency unsteadiness while setting a trajectory for expanded applications.

In this study, we present the design, fabrication, and performance evaluation of the PVDF sensor array in a supersonic flow environment (Mach 2). The calibration and validation tests were performed to assess the sensor’s low-frequency detection capabilities and benchmark its performance against the widely used Kulite^®^ MEMS sensors. The results highlight the PVDF sensor array’s potential as an effective alternative for unsteady wall-pressure measurements in SWBLI studies, with future work planned to extend testing across a broader frequency range.

## 2. Methodology: Processing and Characterization

### 2.1. Material Properties and Design

**Figure 2:**
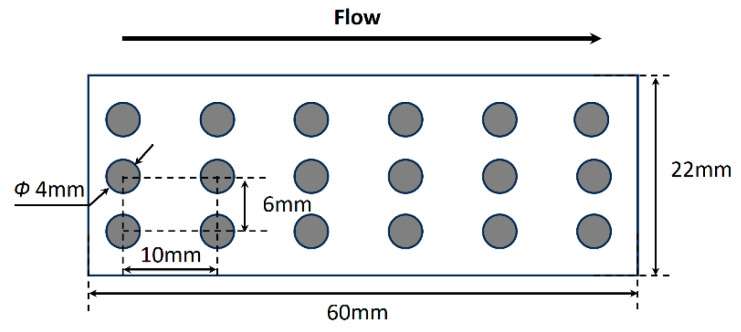
Schematic of the design of sensor elements.

The flexible sensor array was fabricated using screen printing, as shown in Figure 2. A 110 μm-thick PVDF foil (PolyK^®^) (PolyK Technologies, LLC, 2124 Old Gatesburg Road, State College, PA, USA) served as the piezoelectric sensing material (Table 1). The electrode nodes, each 4 mm in diameter, were printed onto the PVDF foil in a 3 × 6 configuration. Nodes were spaced 6 mm apart within each row and 10 mm apart in each column (Figure 2).

### 2.2. FEM Simulation in Frequency Domain

**Figure 3:**
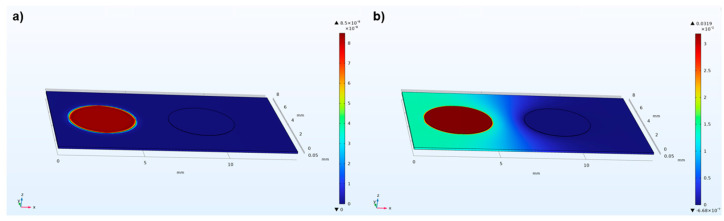
(**a**) Displacement (mm) of PVDF film caused by 1 kPa pressure vertically to the left sensor electrode at 1 kHz, and (**b**) the electric potential caused by the piezoelectric effect of the PVDF film.

**Figure 4:**
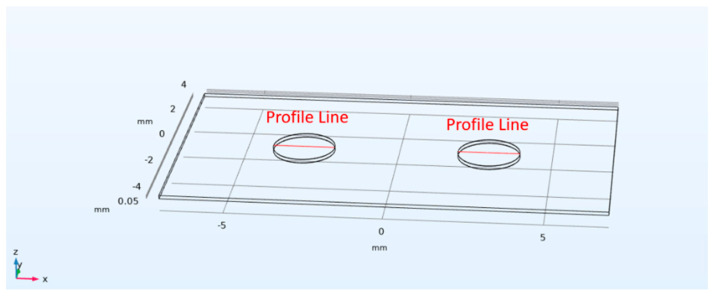
Defined profile lines on the top of the Ag electrodes printed on PVDF film.

**Figure 5:**
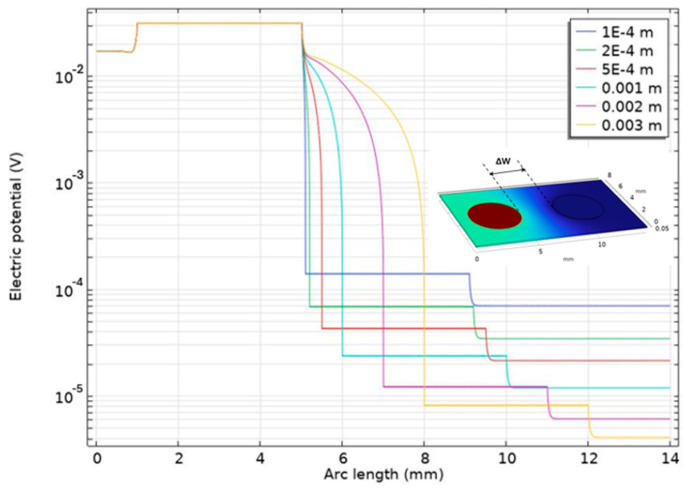
Simulation of signal crosstalk between two adjacent sensor elements at varying distances ΔW.

**Figure 6:**
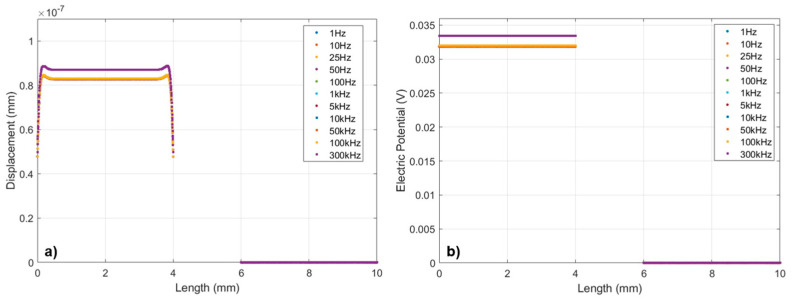
(**a**) The displacement (mm) on the profile lines of FEM simulation in frequency domain analysis, and (**b**) the electric potential caused by the displacement in frequency domain analysis.

The FEM simulation of the PVDF piezoelectric sensor elements was carried out by using COMSOL© Multiphysics 6.2. The model consists of a PVDF piezoelectric film (110 μm) and silver ink electrodes (10 μm) screen-printed in mirrored positions on its top and bottom surfaces. The silver electrodes on the top surface of the PVDF film are defined as the anode, while the other on the bottom is defined as the cathode. Due to the limitations of computational capacities and simulation time, the model was simplified, and only two adjacent sensor element pairs (with a center-to-center spacing of 6 mm) were simulated. A 1 kPa pressure load was applied vertically on the left silver anode. Figure 3a,b shows the deformation/displacement and potential distributions of the model induced by a 1 kHz pressure load, by defining a Profile Line on the surface of the silver anodes (as shown in Figure 4), Figure 5 illustrates the impact of distance Δ*W* between adjacent sensor elements on the output signal crosstalk under identical boundary conditions. Furthermore, Figure 6a,b represents the deformation/displacement and the electric potential of the silver anodes (including the adjacent electrode element) in the model at varying frequencies.

Figure 5 shows the impact of sensor distance ΔW, on the output signal crosstalk under a defined load of 1 kPa and the frequency of 1 kHz at the sensor element on the left side. The output potential of the loaded sensor element on the left is 0.031 V. As the sensor distance increases, the crosstalk effect on the unloaded sensor element on the right gradually diminishes. With a 0.1 mm distance, the output potential of the right sensor element is $1.5\cdot 10^{-4}$ V, which is 0.48% of the output potential of the left sensor element. At 2 mm of distance (the design dimension mentioned above), as indicated by the purple line in Figure 5, the output potential of the right sensor element is $1.3\cdot 10^{-5}$ V, approximately 0.042% of the output potential of the left sensor element. Therefore, at the sensor design dimension used in this work, signal crosstalk can be considered negligible.

Figure 6 shows the curves of deformation/displacement and the potential of the silver anodes as a function of load pressure frequency in the finite element frequency domain analysis. Due to our target frequency range up to 200 kHz, the simulation was defined in the frequency range up to 300 kHz.

In Figure 6a, the displacement profile illustrates deformations of the PVDF film under varying frequencies of applied pressure. As frequency increases, the displacement becomes more complex, indicating that the dynamic mechanical response of the film is frequency-dependent. Figure 6b shows the electric potential distribution caused by the piezoelectric effect. As expected, an increase in frequency alters the electric potential, as the strain-induced charge generation by the piezoelectric effect changes with deformation/displacement.

**Figure 7:**
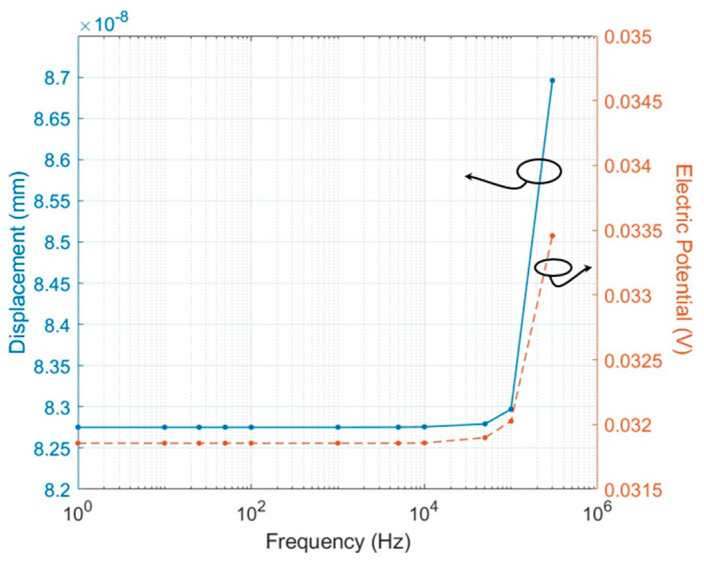
Spectra representation of displacement (mm) and electric potential (V) in frequency domain up to 300 kHz.

Figure 7 shows the relationship between frequency and both displacement and voltage. The curves indicate that both displacement and electric potential evolved with increasing frequency, and there appears to be a threshold where the responses increased obviously. In the frequency range up to 1 kHz, the mechanical deformation/displacement of the model remained constant at $8.27\cdot 10^{-8}$ mm, with a piezoelectric response voltage of 0.031 V. Between 1 kHz and 50 kHz, both displacement and potential response show a slight increase with frequency. From 50 kHz to 300 kHz, the displacement and potential responses exhibit a linear upward trend, reaching $8.69\cdot 10^{-8}$ mm (0.033 V) at 300 kHz, about +5% compared to the response in the low frequency range. This behavior suggests that the PVDF sensor has resonant frequency in the test range [32,33].

The analysis of Figure 7 supports the hypothesis that, at higher frequencies, the deformation/displacement of the PVDF film increases in the frequency domain. This finding is due to the dynamic characteristics of the PVDF material, where its mechanical and piezoelectric properties interact with the frequency of the applied pressure load. As frequency increases, the inertia of the film can lead to more pronounced oscillatory behavior, increasing the deformation until a certain resonant frequency is reached. Furthermore, Figure 6 suggests that the electrical response of the PVDF sensor can behave similarly to a high-pass filter, where, at higher frequencies, the parasitic capacitance and resistance of the sensor become more significant. As frequency increases, the impedance of the capacitive elements decreases, allowing more current (or voltage in this case) to pass through, effectively filtering out low-frequency signals. In summary, the PVDF piezoelectric film’s displacement increases with frequency due to its resonant mechanical behavior. Additionally, at high frequencies, the parasitic capacitance and resistance form a high-pass filter, with the cutoff frequency dependent on the material’s intrinsic properties and the sensor’s electrical design. Notably, the results in Figure 6a,b also indicate that, for the metal electrodes, which have a radius of 2 mm, printed on a 110 μm thick PVDF film, with an edge-to-edge distance of 2 mm between adjacent electrodes, their resulting sensor crosstalk effect in frequency range up to 300 kHz is negligible. These results highlight the sensor’s ability to capture a wide range of unsteady flow phenomena, from low-frequency pressure oscillations to high-frequency turbulence-induced fluctuations.

### 2.3. Processing and Its Electrical Circuit

**Figure 8:**
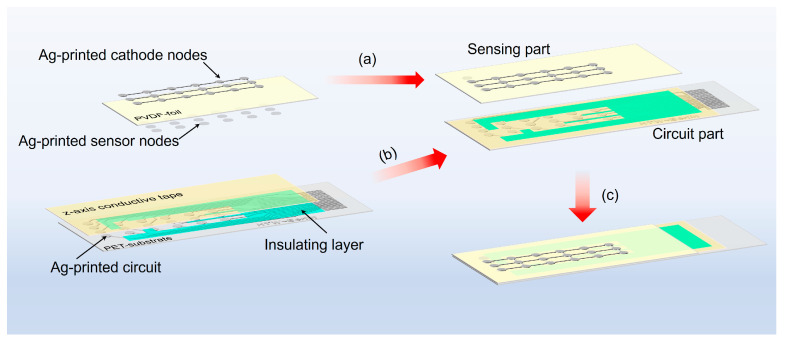
Illustration of 3D-integrated thin-film flexible PVDF sensor array: (**a**–**c**) show the process steps.

Although PVDF sensors offer benefits such as lightweight, flexibility, good ductility, and ease of processing, the high density of sensor elements introduces several challenges, including increased signal line area, crosstalk between signal lines, and potential signal attenuation due to the signal line length. These factors limit the overall sensitivity and stability of the PVDF sensor elements. To address this issue, we propose an innovative assembly method where the sensing and circuit parts are processed separately and then assembled, as shown in Figure 8. For the sensing part, the sensor cathode nodes were printed on the top side of the PVDF film using silver conductive ink Bectron^®^ CP 6667 (ELANTAS Europe, Grossmannstr. 105, Hamburg, Germany). After curing at 50 °C in an oven for 30 min, followed by 1 h of room temperature curing and cooling, the sensor anode nodes were printed on the backside of the PVDF film. The printed sensor anodes also underwent a 50 °C curing process, followed by room temperature curing and cooling, completing the fabrication of the sensing layer as shown in Figure 8a.

For the circuit part, the signal circuit was printed using silver conductive ink on a 110 μm-thick polyethylene terephthalate (PET) film, which is non-piezoelectric and serves as an insulating substrate, followed by the same thermal curing process. To protect signal transmission and isolate the conductive circuits from the sensing area, insulating ink DP 8442 (the green layer in Figure 8b) was printed over the circuits after the previous layer was cured. The curing process of the insulating ink requires 30 min of UV exposure at 5 mJ/cm². Subsequently, 3M^TM^ 9703 z-axis conductive tape (St. Paul, MN, USA) was applied on top of the circuits and their insulating layer to ensure a reliable electrical connection with the sensing part, as shown in Figure 8b. Finally, the sensing and circuit parts were bonded together to create a 3D-integrated thin-film flexible PVDF sensor array, as shown in Figure 8c.

**Figure 9:**
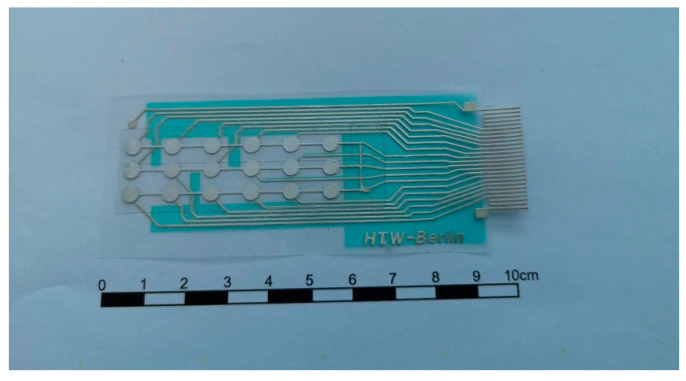
Images of PVDF sensor array with 4 mm sensor elements.

Figure 9 shows an image of the processed sensor array with 4 mm diameter sensor elements. The cathode of the sensor array was connected to the GND pad of the electric circuit on the PET substrate, ensuring that all cathode nodes shared the same electrical potential of 0 V.

**Figure 10:**
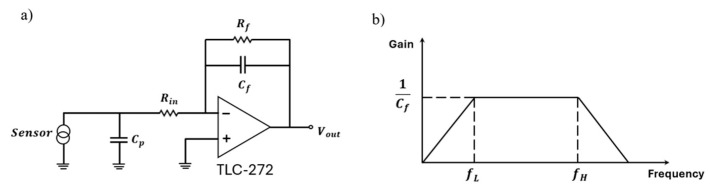
(**a**) The charge amplifier circuit for the PVDF piezoelectric sensor array, and (**b**) the band-pass behavior of the charge amplifier circuit [31,34].

The charge amplifier was constructed using a TLC-272 from Texas Instruments, Dallas, TX, USA, with a feedback resistance $R_{f}=100\;M\Omega$ and a feedback capacitance $C_{f}=100\;\mathrm{pF}$ connected to the inverting input terminal. An input resistance $R_{in}=56\;\Omega$, providing overcurrent protection, was placed between the piezoelectric sensor and the inverting input terminal, as depicted in Figure 10a. This resistance also served as electrostatic discharge (ESD) protection, as indicated in the TLC-272 datasheet [34].

The charge amplifier circuit functions as a band-pass filter, with a low-frequency cutoff at

(1) $$f_{L}=\frac{1}{2\pi R_{f}C_{f}},$$

and a high-frequency cutoff at

(2) $$f_{H}=\frac{1}{2\pi R_{in}C_{p}}.$$

Here, $C_{p}$ includes the sensor’s internal capacitance, cable capacitance, and any other parasitic capacitances in parallel with the sensor.

In this setup, $C_{p}$ is approximately 10 pF, resulting in $f_{L}=15.9\;\mathrm{Hz}$ and $f_{H}=2.84\;\mathrm{GHz}$, as presented in Figure 10b. The data acquisition (DAQ) system NI-USB 6353 from National Instruments™, Austin, TX, USA was used to obtain power spectral densities (PSDs) up to 100 kHz at a sample rate of $f_{s}=200\;\mathrm{kHz}$ with *N* = 200,000 samples collected.

## 3. Calibration

**Figure 11:**
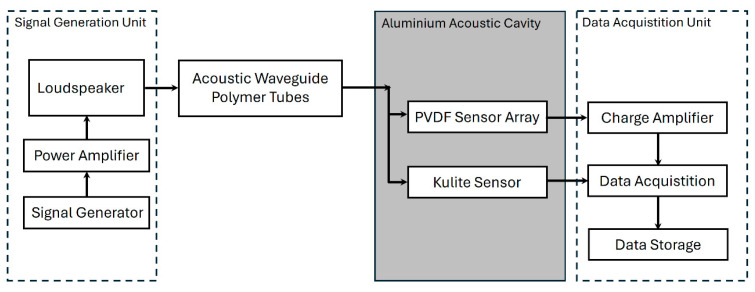
Block diagram of calibration setup.

Ensuring the reliable performance of PVDF piezoelectric sensors, especially at low pressures, requires precise calibration. The acoustic pressure calibration setup is essential for calibrating PVDF thin-film piezoelectric sensor arrays under low-pressure conditions. The key feature of this setup is that it ensures the PVDF sensor array receives a uniform and consistent pressure distribution at the specified sound frequency within the sound pressure detection cavity. The acoustic pressure test setup generated controlled acoustic pressure waves, which were applied to the PVDF sensor array to calibrate and evaluate its performance in comparison with a Kulite sensor under identical conditions, as shown in Figure 11.

**Figure 12:**
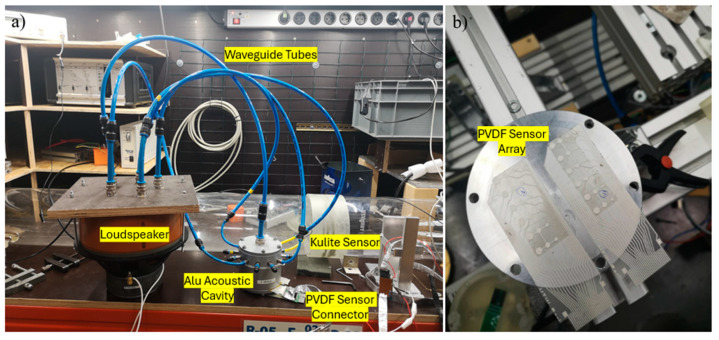
Setup of the sound pressure calibration test equipment (**a**) and the PVDF sensor array test samples (**b**).

In the signal generation unit, a 25 Hz acoustic signal was amplified and transmitted through a loudspeaker, then directed via five plastic acoustic wave conduction tubes positioned on its top cover into an aluminum sound pressure detection cavity, as shown in Figure 11. The plastic acoustic wave conduction tubes have a diameter of 2 cm and a length of 50 cm. Of the five tubes (colored blue in Figure 12), one was directly connected to a central through-hole at the top of the aluminum acoustic cavity, while the other four were connected to equidistant through-holes on the cavity’s sides. These through-holes were located 2 cm from the top surface of the acoustic cavity. The PVDF sensor array was secured at the bottom of the acoustic cavity and sealed with O-rings to ensure airtightness and prevent pressure leakage. A Kulite sensor was also mounted on the side of the cavity, as shown in Figure 12.

At room temperature, with the speed of sound in air c=343 m/s, the wavelength λ of the sound in the tubes is calculated as

(3) $$\lambda =\frac{c}{f}=\frac{343\;m/s}{25\;\mathrm{Hz}}=13.72\;m.$$

According to [35], the resonance frequency $f_{n}$ for the propagation of mechanical waves in a closed-ended tube is calculated as

(4) $$f_{n}=\frac{n\cdot c}{2L},$$

where n is the resonance mode, c is the speed of sound in air, and L=0.5 m  is the length of the tube.

For the first mode n=1, the cutoff frequency $f_{cutoff}$ is calculated as follows:

(5) $$f_{cutoff}=\frac{1\cdot 343\;m/s\;}{2\cdot 0.5\;m}=343\;\mathrm{Hz}$$

This cutoff frequency is much higher than the driven frequency of 25 Hz, indicating that the sound propagates in the fundamental mode without any cutoff or higher-order transmission modes. Therefore, the acoustic pressure distributions and velocity fields in the fundamental mode are uniform across the cross-section. Over the length of the tubes, the sound wave may undergo phase change, but the long wavelength ensures that this effect is minimal. The phase shift Δ∅ over the length *L* is given by the following:

(6) $$\Delta \emptyset =\frac{2\pi L}{\lambda }=\frac{2\pi \cdot 0.5\;m}{13.72\;m}\approx 0.229\;\mathrm{radians}.$$

This mathematical analysis confirms that the sound wave will primarily propagate in the fundamental mode with negligible influence from the tube’s geometry at the specified frequency and dimensions.

**Figure 13:**
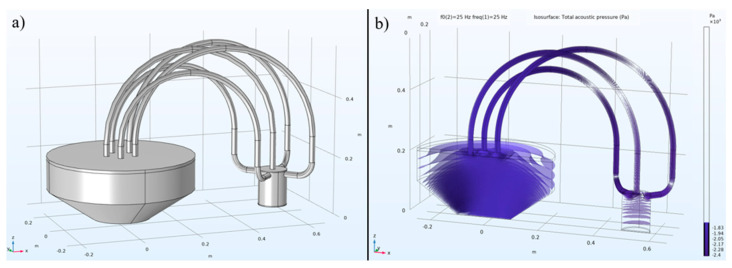
Geometric modeling of the sound pressure calibration test setup (**a**), and the pressure contour distribution (**b**).

The physical bending of the plastic tubes and the mismatch in acoustic signal propagation (e.g., from a narrow waveguide to a wider one) could result in an uneven final sound pressure distribution. To address this issue, we utilized FEM simulations using COMSOL© Multiphysics, as shown in Figure 13. To reduce the number of mesh elements and the computational load, we implemented symmetrical modeling (Figure 13a) and conducted sound pressure simulations on only one half of the mirror-symmetrical structure (Figure 13b).

**Figure 14:**
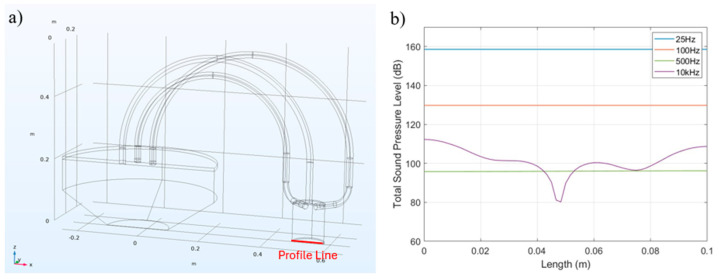
Schematic presentation of the defined profile line in acoustic simulation (**a**), and (**b**) the total sound pressure level (dB) on the profile line at frequency sweep.

To further investigate the distribution of the acoustic pressure field on the bottom surface of the aluminum acoustic cavity, we defined 10 cm profile lines on the cavity’s bottom surface (as shown by the red lines in Figure 14a and conducted simulations with acoustic signals at various frequencies. The results showing the total sound pressure level (dB) are presented in Figure 14b). The results indicate that, within the low-frequency range (≤500 Hz), the pressure field distribution on the bottom surface of the acoustic cavity is uniform. This result implies that each sensor element in the PVDF sensor array receives consistent sound pressure during calibration. However, as the frequency of the acoustic signal increases, the sound pressure is affected by transmission modes and mechanical bending of the tubes, leading to a non-uniform pressure distribution across the PVDF sensor array, as shown in the total sound pressure level (dB) of the 10 kHz sound signal in Figure 14b. In our 25 Hz low-frequency sound pressure calibration platform, the sound pressure measured by the PVDF sensors at each sensor element remained consistent.

**Figure 15:**
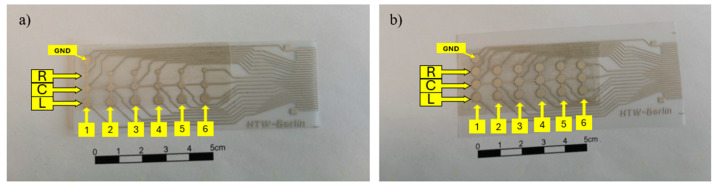
(**a**) Heterogeneous and (**b**) homogeneous sensor arrays.

During the calibration characterization, we utilized both homogeneous and heterogeneous sensor arrays. As shown in Figure 15a, the heterogeneous sensor array consisted of three groups of sensor elements with different diameters arranged along the y-axis into three columns labeled “L” (left), “C” (center), and “R” (right). In this setup, the sensor elements in the L column were circular pads with a diameter of 4 mm, those in the C column had a diameter of 3 mm, and those in the R column had a diameter of 2 mm. In Figure 15b, the sensor elements were also organized into three columns; however, in this array, all nodes were uniform, with a diameter of 4 mm. The sensor elements were further divided along the x-axis into six parallel rows, labeled 1 through 6. The piezoelectric signals from all sensors were collected at each sensor element, transmitted through an interface to the charge amplifier module, and then sent to the DAQ for data conversion and processing.

**Figure 16:**
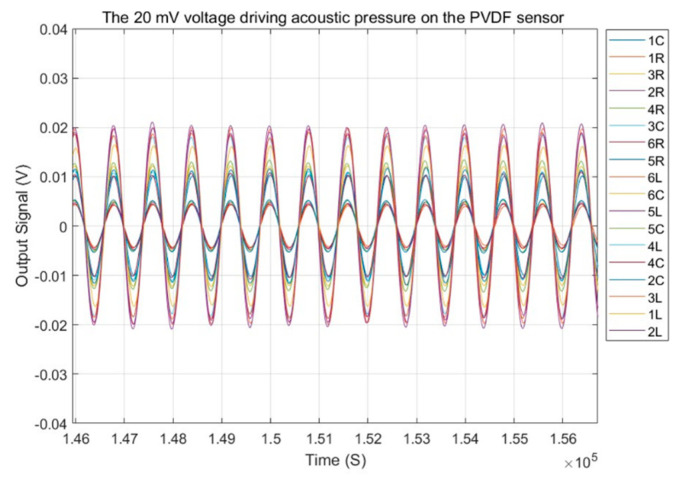
Sensor response of the heterogeneous PVDF sensor array in time domain.

During the signal processing stage, the amplified time-domain voltage signals from the sensors were filtered through a custom-designed IIR Butterworth band-pass filter with cutoff frequencies at 10 Hz and 40 Hz. The sampling rate is 20 kHz. Figure 16 presents the time-domain responses of the 18 sensor elements in the heterogeneous sensor array after filtering. In this figure, 1C represents the first-row sensor elements in the center column, 1L the first-row sensor element in the left column, and 1R the first-row sensor element in the right column. The figure demonstrates that, due to the consistent pressure distribution of the sound wavefront on the sensor surface, there is no phase shift between the time-domain signals. The variations in response are attributed to the different sizes of the sensor elements.

**Figure 17:**
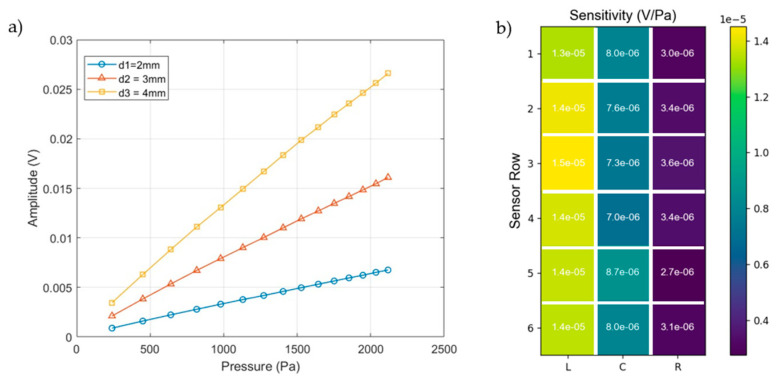
(**a**) Amplitude responses of sensors of different sizes as the driven voltage from the acoustic wave generator increases, and (**b**) sensitivities of each sensor element in the heterogeneous array at a constant pressure of P=240 Pa.

Figure 17 illustrates the output responses of the heterogeneous sensor array. In Figure 17a, the amplitude response of sensor elements of varying sizes is shown as the sound pressure from the acoustic wave generator increases linearly. As the input from the acoustic wave generator increases, so does the sound pressure amplitude (P) on the sensor surface. The x-axis (P) represents the input acoustic pressure in pascals (Pa), while the y-axis represents the voltage response output by the charge amplification circuit in volts (V). This figure highlights how the voltage response changes with input pressure for three sensor sizes (d1 = 2 mm, d2 = 3 mm, d3 = 4 mm). As input pressure increases, all sensors exhibit a corresponding rise in output voltage, with larger sensors generating higher voltage responses. The largest sensor (4 mm) shows the highest output, producing a signal approximately four times greater than that of the smallest sensor (2 mm) at P = 2117 Pa (input acoustic pressure of 300 mV power input), indicating a proportional relationship between size and response. Figure 17b highlights the sensitivity of 18 sensor elements in the array under a driving voltage of P = 240 Pa (input acoustic pressure of 20 mV power input). The 4 mm sensors (L1 to L6) exhibit higher sensitivity values in the range of 14 μV ± 6%/Pa compared to their average value. The 3 mm sensors (C1 to C6) have their sensitivities in the range of 7.7 ± 9% μV/Pa, while the 2 mm sensors (R1 to R6) show lower sensitivities in the range of 3.2 ± 10% μV/Pa.

**Figure 18:**
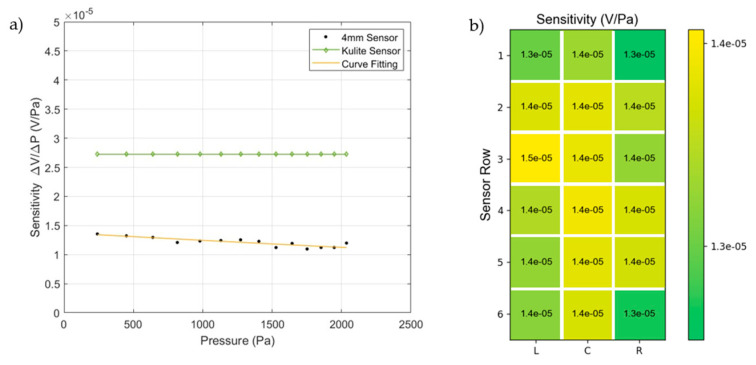
(**a**) The sensitivity of PVDF sensors (4 mm) and the Kulite sensor vs. increasing pressure, and (**b**) the sensor sensitivity of each sensor element of the test homogeneous PVDF sensor array at pressure P=240 Pa.

Figure 18a compares the sensitivity of the 4 mm PVDF sensor elements to the Kulite sensor across different pressure levels. The Kulite sensor exhibits consistent sensitivity throughout the range, with a value of 27.241 μV/Pa. In contrast, the 4 mm PVDF sensor, marked by black dots, shows sensitivities between 10.96 μV/Pa and 13.55 μV/Pa, with a slight decline as pressure increases. Its average sensitivity is 12.1 μV/Pa, with an error margin of ±10%. Unlike the PVDF sensor, the Kulite sensor maintained higher and more stable sensitivity. The PVDF sensor’s performance variation could be due to its piezoelectric material’s response to ambient temperature or environmental factors during assembly.

Figure 18b displays the sensitivity distribution across different positions in a 4 mm PVDF sensor array at 240 Pa acoustic pressure. Sensitivity values range from 12.82 μV/Pa to 14.52 μV/Pa, with the highest sensitivity at position 3L and the lowest at position 1R. The average sensitivity is 13.86 μV/Pa, with an error margin of ±8%. This error may be due to environmental factors in the laboratory and equipment errors during sensor assembly. Despite applying high pressure to exhaust the gas microbubbles before calibration, microbubbles may remain trapped at the sensor’s edges, affecting the sensitivity of sensors located there.

## 4. SWBLI Study in Mach 2 Wind Tunnel

### 4.1. Experimental and Measurement Setup

The PVDF sensors were positioned beneath the separation bubble to capture pressure fluctuations in the interaction region. Kulite sensors were also installed for comparison, offering a benchmark for the PVDF sensor performance.

The experiments were conducted in the supersonic wind tunnel at the Technical University of Berlin. The supersonic wind tunnel was driven up to 16,000 rpm by a DC-motor with 400 kW power, as Figure 14 shows. The centrifugal compressor of the wind tunnel drew the air from outside into the tunnel, and the air was dried as it flowed through the silica gel drying chamber.

**Figure 19:**
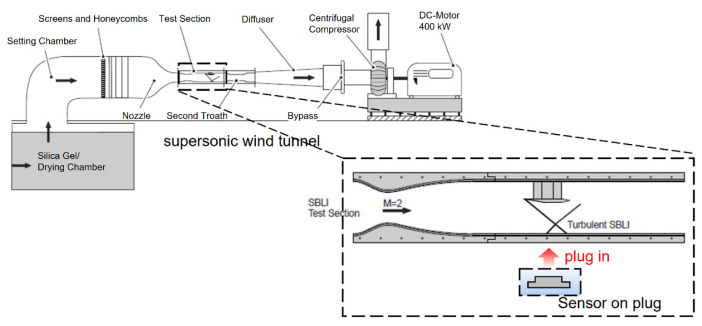
Schematic representation of the supersonic wind tunnel present at the Technical University of Berlin with a focus on the turbulent SBLI test section [28].

**Figure 20:**
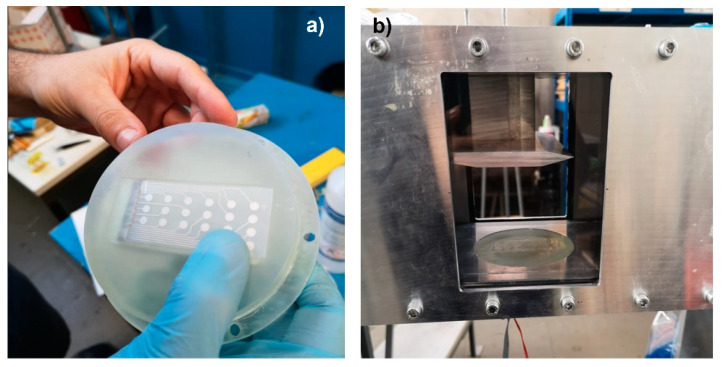
Installation of pressure sensor on plug (**a**), and (**b**) the plug was embedded in the test section in the wind tunnel.

The air flow reached the Laval nozzle, which accelerates the air up to Mach number 2 at the entrance to the test section. A wedge shock generator with an angle of *Φ* = 10° was installed at the top of the test section creating an oblique SWBLI. As shown in Figure 19, a plug was employed as a platform at the bottom of the test section for installing sensors to capture physical parameters in the test section. The PVDF sensor array was glued flat on the plug. As shown in Figure 20a,b, its signal conductive wires passed through the reserved slot on the plug board and connected with the electric circuit part from the backside of the plug. It is worth noting that the embedded plug must be installed into the wind tunnel with high tightness to avoid the acoustic noise from the connection gaps around the plug edges. The configuration of the wind tunnel is shown in Table 2.

**Figure 21:**
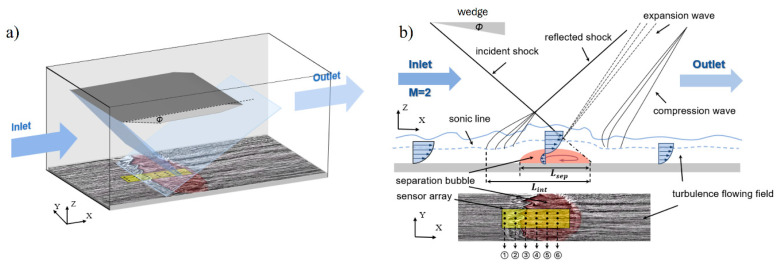
Schematic of (**a**) shock wave/boundary layer interaction with Mach 2.0 flow intake in 3d-presentation, and (**b**) the sensor mounting in the measurement.

When the Mach 2 flow inside the wind tunnel test section meets the shock generator, a shockwave is formed. The resulting oblique shockwave impinges on a fully turbulent boundary layer with a thickness of $\delta_{0}=7.2\;\mathrm{mm}$ on the surface of the wind tunnel floor, which was also observed experimentally by Rohlfs et al. [28,31]. As depicted in Figure 21a, this interaction creates a strong adverse pressure gradient, causing the boundary layer to separate. This separation results in a bubble of reversed flow with a characteristic length $L_{sep}$. The curvature of this bubble induces converging compression waves that merge into separation shock, interacting with the incident shock to form the characteristic λ-foot structure [36,37]. Another crucial parameter for subsequent analysis is the interaction length, $L_{int}$. This parameter is defined as the distance between the extrapolated impingement point, $x_{imp}$ (based on inviscid shock theory) and the interaction onset, $x_{0}$, where the wall pressure begins to increase [1,4,6]. To investigate unsteadiness of this kind of flow, the piezoelectric sensor is placed beneath the separation bubble, and the sensor is defined by the row marked ① to ⑥ in Figure 21b.

In the field of shock wave/boundary layer interaction research, Kulite pressure transducers have gained wide recognition as a prominent technique for measuring wall pressure [18,28,31]. In this study, the Kulite sensor XCQ-062 (Kulite Semiconductor Products, Inc., One Willow Tree Road, Leonia, NJ, USA) [38] was employed as a reference and subjected to the same supersonic conditions. Unlike the PVDF thin-film pressure sensor, Kulite sensors are expensive and must be flush-mounted in the test plug, which entails drilling a hole at each detection position before installation. This requirement arises from the cylindrical packaging of the Kulite sensor, which is 1.7 mm in diameter and 9.5 mm in length. The intricacies associated with this installation process are regarded as one of the primary drawbacks of utilizing Kulite sensors in shock wave/boundary layer interaction study.

### 4.2. Results

Wind tunnel tests at Mach 2 yielded real-time voltage responses from the sensors, which were processed using a high-order FIR filter. After the spectral analysis process, the resulting PSD data provided insights into the frequency characteristics of the unsteady flow. The PVDF sensors detected pressure fluctuations across different positions within the separation and reattachment regions, capturing the energy distribution within the flow field (Figure 21) [15,28,31,39,40].

**Figure 22:**
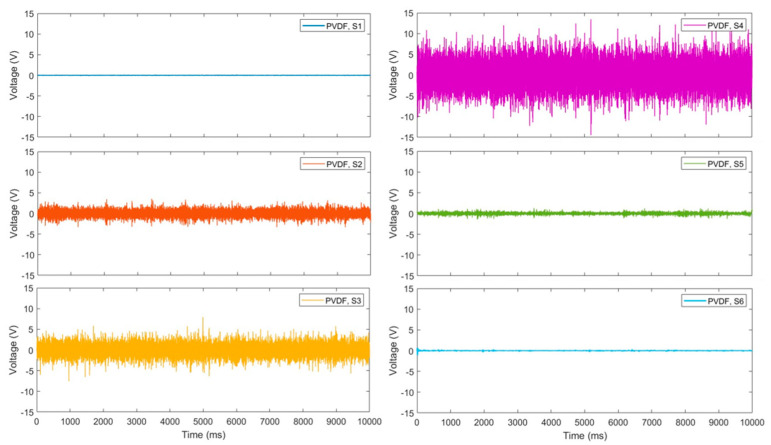
PVDF sensor responses at 6 positions in time domain.

Through wind tunnel tests at a wind speed of Mach 2, we obtained the voltage response of the sensors (S1 to S6) in time domain, as shown in Figure 22. To further filter the low-frequency noise, the signal was processed through a designed high-order FIR (finite impulse response) high-pass filter using a Kaiser window function. The Kaiser window function adjustment parameter was β=5. The FIR filter has an order of 500 and a cutoff frequency of 70 Hz.

**Figure 23:**
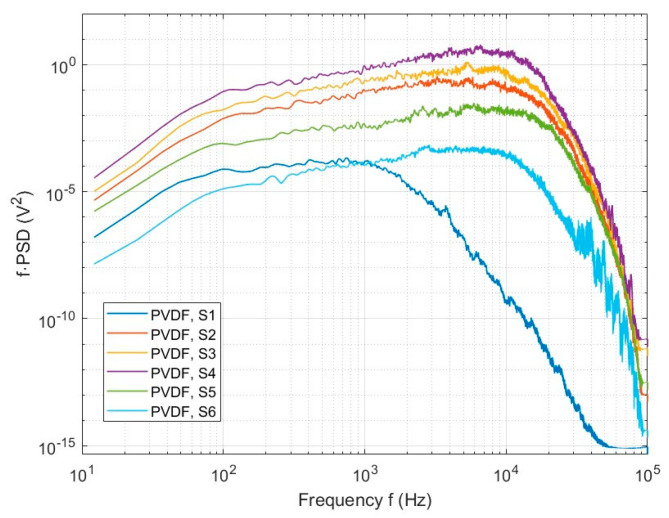
Value f·PSD of 6 PVDF sensors S1 to S6 in frequency domain.

By utilizing the pwelch function in Matlab^®^ R2022b [41], these voltage response data were converted into Power Spectral Density (PSD). In the pwelch function, the Hanning window was used with a window length of 1/20th of the signal length and 50% overlap. Further, to more effectively demonstrate the energy distribution of the signal at different frequencies, particularly the low-frequency unsteadiness, pre-multiplied Power Spectral Density (f·PSD) was used. Figure 23 shows the f·PSD values from sensors along the x-direction in the middle row of the PVDF sensor array, namely, S1 to S6. The plot highlights the variation in frequency responses at different positions within the separation and reattachment regions. The PVDF sensors provide insights into the pressure fluctuations and energy distribution within the flow field.

**Figure 24:**
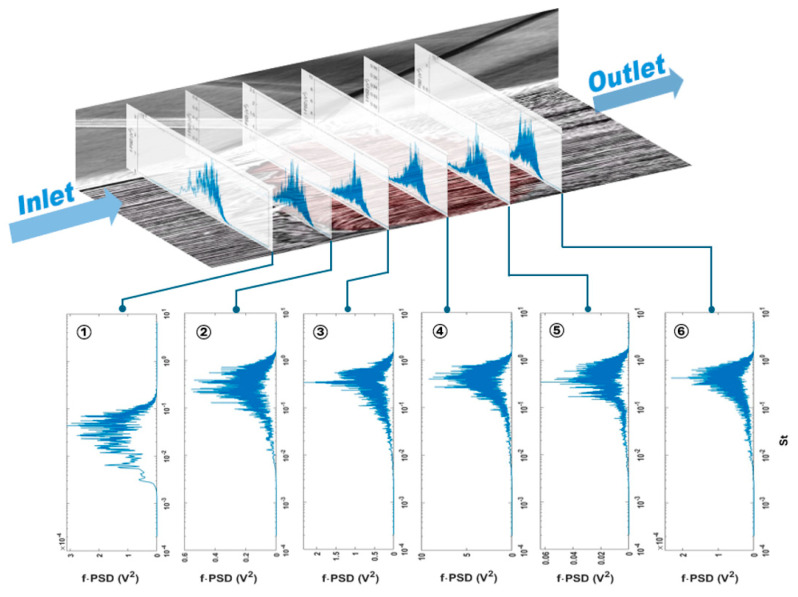
Schematic of the flow field and respective f·PSD spectra along the streamwise direction for the PVDF sensor array.

Figure 24 shows the f⋅PSD values from sensors along the x-direction in the middle row of the PVDF sensor array. The frequency has been normalized by the Strouhal number $S_{t}$ which is defined as $S_{t}=fL_{int}/u_{\infty }$, where f is the frequency, the free stream velocity is $u_{\infty }=512\;m/s$, and the interaction length obtained is $L_{int}=54.5\;\mathrm{mm}$ with the ∅=10° edge shock generator [28,31]. Referring to the parameters from a paper based on the same wind tunnel experiment [28,31,42], the normalized position parameter of the sensors, $X^{*}=\left(x_{i}-x_{0}\right)/L_{int}$, was calculated based on the interaction length $L_{int}$.

The provided plots offer significant insights into the energy distribution of the flow across various frequencies and locations within the shock wave/boundary layer interaction (SBLI). Consistent with the established literature, distinct frequency ranges are observed, each categorized into specific zones characterized by unique temporal scales [43,44,45,46].

These zones are as follows:

**Low Frequency Zone (LFZ)**: This zone is captured by sensor S1, and it is situated at the onset of the interaction region, near the foot of the reflected shock at approximately $X^{*}$. It is marked by very low-frequency fluctuations, featuring a broad peak centered around $0.04<S_{t}<0.06$, which indicates large-scale pressure fluctuations due to the low frequency motion of the reflected shockwave.

**Intermediate Frequency Zone (IFZ)**: Captured by sensors S2 to S6 and spanning the interaction region of $0.2<X^{*}<1$, inside this region, the frequency response has a broader range compared to the low frequency zone and exhibits medium-frequency characteristics at $S_{t}\approx 0.5$, which is in between the low frequencies’ fluctuations of the reflected shock foot and the high frequencies of the small-scale turbulence of the supersonic boundary layer. Usually, the IFZ region is associated with the shedding of large-scale coherent structures in the shear layer that develops over the shock-induced separation bubble.

For comparison, we integrated six Kulite sensors in the sensor plug, parallel to the S1 to S6 PVDF sensors. Both the PVDF sensors and the Kulite sensors were tested under the same condition simultaneously.

**Figure 25:**
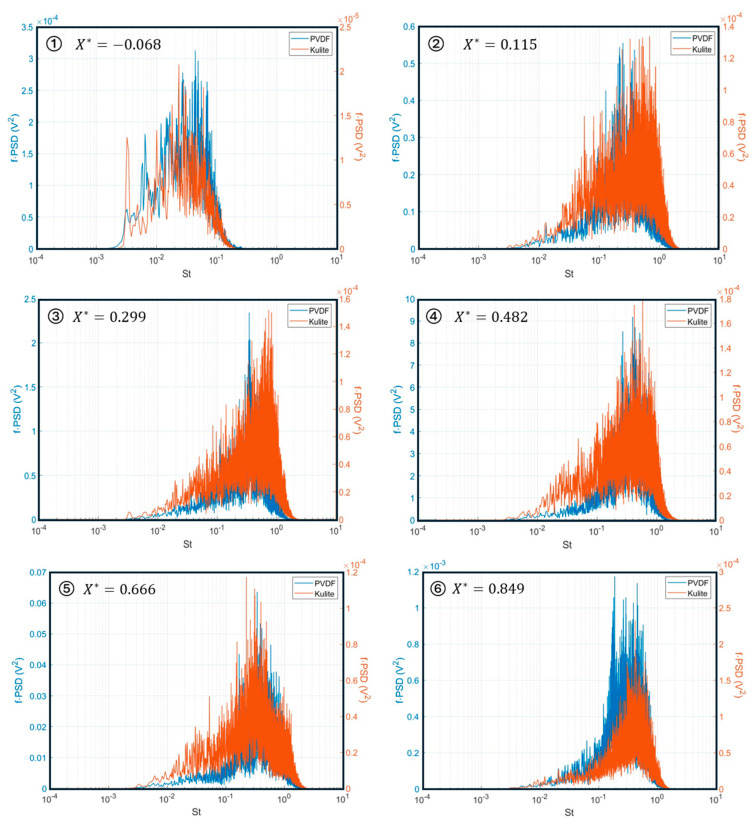
Comparison of values f·PSD vs. Strouhal number $S_{t}$ at six investigation positions ① to ⑥ with Kulite sensors under the same conditions in the wind tunnel.

As shown in Figure 25, the power spectral density f·PSD show that the PVDF sensors exhibit a strong consistency with the Kulite sensors located at the same positions along the x-direction of the air flow. At location ①, the PVDF sensor and the Kulite sensor have very low frequency fluctuations and a broad hump centered in the Strouhal number range $0.04<S_{t}<0.06$. The peaks of the Kulite response at $S_{t}=0.0033$ indicates the main hum noise at 50 Hz. From the location ② to ⑥, both the PVDF sensors and the Kulite sensors exhibit significant narrow humps in the range $0.4<S_{t}<0.5$.

Since the PVDF sensors and Kulite sensors were measured under the same conditions during the wind tunnel experiment, there is no need to consider the time lag between the data. We chose the Pearson’s Correlation Coefficient r to measure the correlation between the normalized power spectral densities of both the PVDF sensor and the Kulite sensor [47]:

(7) $$r=\frac{\sum \left(x_{i}-\bar{x}\right)\left(y_{i}-\bar{y}\right)}{\sqrt{\sum {\left(x_{i}-\bar{x}\right)}^{2}\sum {\left(y_{i}-\bar{y}\right)}^{2}}},$$

where $x_{i}$ and $y_{i}$ are the observed values of the two data sets, and $\bar{x}$ and $\bar{y}$ are the means of the two data sets.

**Figure 26:**
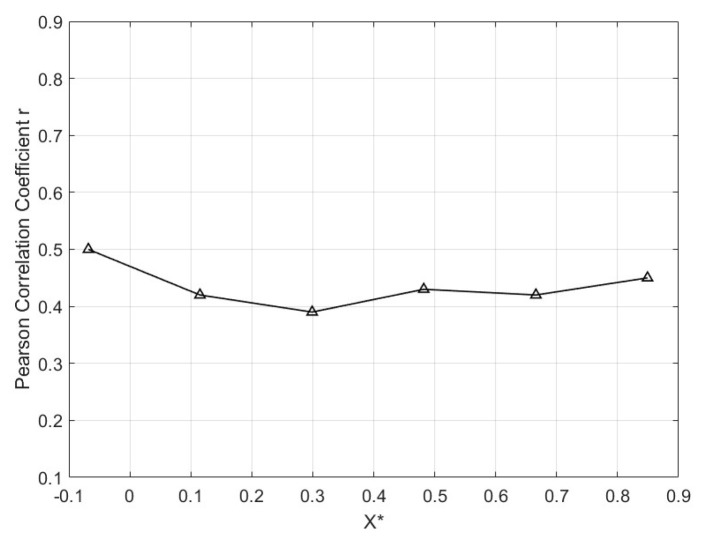
Illustrates the similarity of the pressure signal values received by the two types of sensors at the same location and time.

Comparison with the Kulite sensors showed that the PVDF sensors demonstrated strong consistency in detecting unsteadiness, particularly in the low-frequency range. Pearson’s correlation coefficient analysis indicated moderate positive correlation (0<r<1) between the two sensor types, validating the PVDF sensor array’s effectiveness. As in Figure 26, the Pearson correlation coefficient fluctuates between different $X^{*}$ values but consistently remains between 0.42 and 0.52. This finding indicates that the correlation between sensor variables at different positions or time points is relatively stable. Notably, in sensor region ①, the correlation coefficient is the highest, reaching 0.5. In sensor region ③, the correlation is the weakest, at 0.39. From region ④ to ⑥, the correlation shows an increasing trend. The possible reasons for these differences may be attributed to the varying design size of the sensing transduces, sensitivity, and spectral response resulting from the different sensing mechanisms of piezoelectric (PVDF sensor) and piezoresistive (Kulite sensor) sensors as well. Additionally, the positioning of the sensors within the wind tunnel, measurement environment errors, human error, or even a combination of multiple factors could contribute to these variations.

## 5. Conclusions

Finite Element Method (FEM) simulations were conducted to investigate the PVDF sensor array’s frequency response characteristics, particularly its potential for high-frequency operations up to 300 kHz. The simulations revealed that the sensor exhibits high-pass filter behavior due to the interaction of its capacitive and resistive properties with circuit parasitics. This behavior suggests that the sensor can effectively minimize low-frequency noise while maintaining stability in the high-frequency range, a promising feature for applications that require sensitivity to dynamic pressure fluctuations, such as SWBLI studies.

The current experimental work, however, concentrated on validating the PVDF sensor array’s low-frequency response, which aligns with the primary needs of SWBLI research. Low-frequency unsteadiness is a key factor in SWBLIs, driving phenomena like flow separation and recirculation, making accurate detections within this range essential. By first establishing a reliable low-frequency performance, this study addresses the immediate requirements for SWBLI research while setting a foundation for future high-frequency testing.

In wind tunnel experiments conducted at Mach 2, the PVDF sensor array demonstrated the effective detection of unsteady low-frequency pressure fluctuations. The results were consistent with data from Kulite sensors, confirming the PVDF array’s sensitivity and reliability as a viable alternative for SWBLI applications. These findings underscore the PVDF sensor array’s suitability for low-frequency unsteadiness studies, with its high-frequency potential—indicated by an FEM simulation—reserved for subsequent experimental validation.

This study’s main contributions include the development and initial validation of a PVDF sensor array tailored for low-frequency wall-pressure measurements in SWBLI environments. The FEM simulations suggest that the sensor could maintain stable responses up to 300 kHz, providing a theoretical basis for high-frequency applications. While current experiments focused on low-frequency calibration, future work will extend testing to cover the high-frequency range. This further testing will allow for a comprehensive evaluation of the PVDF sensor array’s operational range and dynamic response under varied flow conditions, enhancing its potential as a versatile tool in broader aerodynamic and aerospace applications, including high-frequency pressure measurements.

Future work will involve controlled testing of the PVDF sensor array under varying temperature and humidity conditions to assess its long-term stability and sensitivity. These tests will help evaluate the robustness of the PVDF material and validate its performance in diverse environments commonly encountered in aerodynamic research.

As an alternative piezoelectric sensing material, PVDF polymers, particularly in their nanofiber form, exhibit remarkable sensitivity to various mechanical stimuli, including pressure, strain, vibration, and motion [48]. This exceptional versatility has enabled their widespread application across diverse sensing technologies, further highlighting the potential of PVDF-based sensors for innovative advancements in the field. 

## Figures and Tables

**Table 1:** Characteristic parameters of PVDF piezoelectric foil [30,31].

Item	Value	Unit
Piezoelectric Strain Constand $d_{33}$	28	pC/N
Piezoelectric Voltage Constant $g_{33}$	0.3	V·m/N
Relative Dielectric Constant $\varepsilon_{r}$	13.5	
Maximum Operating Voltage	>100	V/μm
Tensile Strength $\sigma_{MD}$	$\sim 0.4\times 10^{9}$	$N/m^{2}$
Young’s Modulus $\gamma_{MD}$	$\sim 2.8\times 10^{9}$	$N/m^{2}$
Poisson’s Ratio	0.3	
Density	$1.78\times 10^{3}$	$\mathrm{kg}/m^{3}$
Frequency Range	$10^{-3}\;\sim \;10^{10}$	Hz
Pressure Range	$0\;\sim \;10^{9}$	Pa
Operating Temperature Range	−40 ~ 80	°C
Thickness	110	μm

**Table 2:** Configuration of the wind tunnel for the SWBLI [30].

Item	Value	Unit
Mach Number M	2	
Freestream Unit Reynolds Number $R_{eU}$	$1.5\times 10^{6}$	$m^{-1}$
Boundary Layer Velocity Thickness $\delta_{0}$	7.2	mm
Shock Generator Angle Φ	10	degree
Test Section Length $L_{t}$	500	mm
Cross-Section Dimension	150×150	mm×mm

## Data Availability

The raw data generated and analyzed during this study are not publicly available due to proprietary restrictions. However, aggregated data and detailed methodologies are provided within the article to ensure the reproducibility of the results. For specific inquiries, please contact the corresponding author.
